# Defining the level of evidence for technology adoption in the localized prostate cancer pathway

**DOI:** 10.1016/j.urolonc.2013.10.008

**Published:** 2014-08

**Authors:** Massimo Valerio, Ahmed El-Shater Bosaily, Mark Emberton, Hashim U. Ahmed

**Affiliations:** a Division of Surgery and Interventional Science, University College London, London, UK; b Department of Urology, University College London Hospitals NHS Foundation Trust, London, UK; c Department of Urology, Centre Hospitalier Universitaire Vaudois, Lausanne, Switzerland

**Keywords:** Focal therapy, Health technology assessment, MRI, Prostate cancer, Targeted biopsy, Trials

## Abstract

New technologies in prostate cancer are attempting to change the current prostate cancer pathway by aiming to reduce harms while maintaining the benefits associated with screening, diagnosis, and treatment. In this article, we discuss the optimal evaluation that new technologies should undergo to provide level 1 evidence typically required to change the practice. With this in mind, we focus on feasible and pragmatic trials that could be delivered in a timely fashion by many centers while retaining primary outcomes that focus on clinically meaningful outcomes.

## Introduction

The prostate cancer pathway from screening and diagnosis to treatment has recently been critically questioned in light of level 1 evidence pointing to pathway-related harms that may outweigh the benefits in many men. As a consequence, recent guidelines have recommended limiting the systematic use of prostate cancer screening based on prostate-specific antigen (PSA) testing to avoid overdiagnosis and overtreatment [1], [2], [3], [4]. There is a clear requirement to improve the current therapeutic ratio of the entire diagnostic and therapeutic pathway with novel interventions. As a result, interest has focused on applying multiparametric MRI (mpMRI) in men at risk before biopsy, targeted biopsy based on mpMRI-derived suspicious lesions, and tissue-preserving focal therapy.

Although several academic expert centers currently using these new technologies are attempting to drive the paradigm changes in the prostate cancer pathway, many others remain skeptical. The level and degree of evidence required to tip the adoption curves of these new interventions and technologies to wider dissemination and diffusion is open to debate. Of particular note, many cite the need for high-quality studies, which reduce the risk of internal bias, and maintain that only randomized controlled studies (RCTs) and systematic reviews of RCTs can change practice across the board [5], [6], [7].

Although it is certainly true that there is an acute lack of level 1 evidence in the field of new interventions for localized prostate cancer, it is well known that performing RCTs in surgical sciences is more challenging than in other medical sciences [8]. In the absence of level 1 evidence in surgical sciences, there have been examples of widespread adoption based on high-quality prospective series. For instance, partial nephrectomy in the treatment of small renal masses and minimally invasive treatment for penile cancer are prime examples [9], [10].

The key to using prospective single-arm data is to ensure that such data are of a high quality. In addition to the levels of evidence defined by Sackett, a mechanism to assess the quality of evidence in health technology assessment may be more adequate in surgical sciences [7]. The Grading for Recommendation Assessment Development and Evaluation system considers study quality and estimates the degree to which one can deem an effect size correctly [11]. With this objective, in addition to the study design, the Grading for Recommendation Assessment Development and Evaluation system calculates the quality of evidence considering other important criteria, which can significantly increase or decrease the overall score. These criteria are risk of bias, inconsistency, indirectness, imprecision, and publication bias. Practically, this system considers the external validity and generalizability of the results of a study as important as its internal validity.

In this article, we discuss the optimal evaluations that new technologies in prostate cancer should face to change the current paradigm, and which the wider urological community might accept. In doing so, we focus on feasible (in terms of deliverability) study designs with pragmatic clinically meaningful outcomes that may be based on international recommendations and patient/physician consensus. The articles in this guest edition have focused on a number of elements of pathway change that have been of particular interest in the last 5 years—namely, target detection and target ablation. We address each of these separately.

## Target Detection

### Multiparametric MRI as the first test in the pathway

Many prostate cancers currently detected are clinically insignificant, meaning that they do not have any clinical effect on the individual during lifetime [12]. Currently, the diagnostic pathway for prostate cancer remains heavily reliant on what many accept is a random biopsy process taken under transrectal ultrasound (TRUS) guidance, which has shown several problems owing to its nontargeted nature and susceptibility to systematic sampling errors. In addition, TRUS biopsy is a relatively invasive test that carries the risk of harm to the patient, such as bleeding, discomfort, and sepsis [13]. These risks can increase when TRUS biopsies are repeated, which is often done owing to the poor negative predictive value of TRUS [14].

A test that is able to triage men to identify those with likely clinically significant prostate cancer might have considerable clinical utility [15]. Such a test would be applied when there is an indication for biopsy—usually because the test showed an elevated PSA level—and prior to the biopsy itself. Although many biomarkers are being assessed, imaging in the form of mpMRI may hold the greatest value in this regard [16]. At present, mpMRI seems to demonstrate very promising performance characteristics as a triage tool to exclude clinically significant prostate cancer and select patients who are likely to benefit from diagnosis [17]. Indeed, in prospective expert center series with expert reporters, when preoperative mpMRI showed no suspicious lesion, in 95% of those areas, there was no evidence of disease that many would agree constituted clinically significant cancer on whole-mount step-sectioned histology using radical prostatectomy specimens [18]. At the same time, mpMRI is a noninvasive test, which does not carry the same concerns related to TRUS biopsy complications. Despite these results, mpMRI is still used by only a few experienced centers because the level of evidence cannot be considered of high enough quality to change practice.

Why is there doubt about the literature? Arguably, the current literature is limited by a number of biases. The studies published have mostly evaluated mpMRI after biopsy, which compromises results owing to biopsy artifact, and so the accuracy of mpMRI might have been underestimated. In addition, many groups have limited the correlation of mpMRI with histology to the peripheral zone alone. Furthermore, the reference test is a significant source of bias in itself. Most studies have used radical prostatectomy as comparator, leading to selection of only patients who have cancer and who then choose or are recommended to have surgery. Men with borderline or abnormal PSA, those who had negative result on TRUS biopsy—which might actually be positive or negative if they were to undergo an accurate verification test—or those who opted for other treatments would not be assessed. Furthermore, for diagnostic tests to be deemed reliable, they must be assessed for test-retest reliability and show consistent results in terms of interobserver and interitem variation. Finally, to be deemed valid, diagnostic tests should have face validity and content validity. Face validity is the degree to which the results of a test actually represent the measured values, whereas content validity attempts to verify that the method of measurement (here mpMRI) actually measures what it is expected to measure (benign and malignant prostate tissue).

The Standards for Reporting of Diagnostic Studies (STARD) statement has provided researchers with minimum requirements for reporting studies that evaluate diagnostic tests. The checklist in essence allows researchers to design high-quality study designs [19]. Thus, level 1 evidence for diagnostic tests includes the paired validating cohort design as it overcomes the biases mentioned before and retains both internal and external validity [15]. The paired validating cohort study design includes the index test (under evaluation), the reference test for accuracy validation, and the standard test(s) applied to all participants in the study. All tests are conducted in a blinded fashion by a priori defined standard operating procedures.

The Prostate MRI Imaging Study (PROMIS) was designed to avoid common pitfalls in diagnostic trial design that lead to inaccurate estimation of test accuracy [20], [21]. PROMIS is a multicenter validating paired cohort study where all patients undergo the index test (mpMRI) and then proceed to have the standard test (TRUS biopsy) and the reference test (transperineal prostate mapping with 5-mm sampling density). Template prostate mapping biopsies are the gold standard test for this particular research question and this population, as they have excellent accuracy and can be applied to all men in the study, regardless of the index test outcomes [22].

This paired validating cohort design has several advantages [15]. The trial recruits all patients who have been recommended a biopsy, and each patient is evaluated by the 3 tests; thus, spectrum and selection biases are minimized. In addition, rigorous blinding of each test result is employed as the findings of each test are reported centrally and not released to patients or to other health care professionals within any of the centers. Therefore, patients and all clinicians, including radiologists, urologists, and pathologists, will remain blinded to the results until all tests have been reported and those reports locked. This would eliminate workup bias or any effect that knowledge of the imaging may have on the manner in which the biopsies are conducted. Finally, standard operating procedures detailing the method of conducting and reporting all 3 tests avoid variation in the technique from one clinician to another. These standards are especially helpful in the multicenter setting of PROMIS. On the contrary, the multicenter setting also contributes to the strength of this design as it is simply not enough to evaluate the accuracy of mpMRI alone in 1 specialized center (one of the problems with the current literature), but it is more important to determine how feasible it would be to use an mpMRI-based pathway across a health care system (scanner availability, reporter training/expertise, and interobserver variation).

### Biopsy of the target lesion

Currently, the diagnostic pathway for prostate cancer is not influenced by the location of prostate cancer foci in a particular patient but rather deploys the needles to areas of the prostate likely to harbor disease (predominantly the peripheral zone). As a result, some areas are systematically undersampled such as the base and apex as well as large areas of the peripheral zone itself [23].

A systematic review comparing the accuracy of an MRI-derived targeted biopsy with standard TRUS biopsy for the detection of clinically significant disease has recently shown that the use of a targeted approach decreases the number of biopsies and at the same time improves cancer risk stratification [24]. As a result, this new strategy may improve the selection of patients for active management, may decrease the number of biopsy-related adverse events by decreasing the number of samples taken, and may confer a more accurate diagnosis. However, this systematic review stated that most of the studies included were noncompliant with the standards described in the STARD statement and the evidence could not be considered of a high quality [19]. Clearly, a standardized approach to conducting and reporting these studies is needed.

Attempting to improve the quality of evidence, a recent statement has guided researchers with recommendations on how to report the results of series comparing standard biopsy to targeted biopsy, and in effect guide the conduct of such studies in a prospective manner. The Standards of Reporting for MRI-targeted Biopsy Studies of the prostate recommendations have provided a list of preferred terminology to use, a detailed checklist of items, and the trial design information that should be detailed when reporting such studies [25]. It also emphasized the need to report the conduct of the diagnostic mpMRI specifically regarding the scanner details and the sequences used. The biopsy procedure itself should also be reported explaining the method used to obtain the samples, whether the operator was blinded to the diagnostic images, and whether standard or targeted biopsies had been taken first. Furthermore, researchers were also advised to separately report in a 3×3 table the results of standard versus targeted biopsy regarding presence of clinically significant disease, of clinically insignificant disease, and any cancer. These recommendations should improve study quality and facilitate the progressive building of robust evidence by allowing investigators to collate and meta-analyze data from a number of groups.

It is also important to emphasize that to provide level 1 evidence of superiority of a new diagnostic modality, the methodology should be rigorous as per the STARD statement [19]—poor studies simply reporting to the points of the Standards of Reporting for MRI-targeted Biopsy Studies do not ensure that the study is of good quality [15].

## Target ablation

### Tissue-preserving focal therapy

The UK Medical Research Council recently updated its guidelines for evaluating complex interventions, such as surgical procedures [26]. These recommendations include the consideration of alternative designs to RCTs, the use of pilot studies to guide the design of effectiveness trials, the use of experimental designs when possible to boost recruitment and study completion, and finally the use of several clinically meaningful outcomes, with possible subgroup analysis if necessary with larger samples. This may all sound intuitive and logical, but this document was a response to the acknowledgment that the evaluation of complex/surgical interventions is more challenging than the evaluation of drugs for several reasons. First, the effectiveness of one intervention is highly dependent on the skills not only of the surgeon but mainly of a team composed of many health care professionals, each playing an essential interconnected role. Thus, the generalizability of the results of 1 surgical team might be very limited. Second, traditional RCTs comparing a new intervention to a standard one are very difficult to deliver both because surgeons may not be equally skilled in the 2 interventions evaluated and because the patient and surgeon may have a more strong preference for one of the procedures (lack of patient and physician equipoise). Finally, the use of placebo and the employment of effective blinding are rarely feasible or ethical in surgery.

In an attempt to both highlight these differences and advise researchers, funding agencies, and regulatory bodies on how to evaluate surgical innovations in a robust manner, the Balliol Collaboration has recently released recommendations, which are reported in the Innovation, Development, Exploration, Assessment, and Long-term study (IDEAL) guidelines [27], [28], [29], [30]. Essentially, the assessment of surgical innovation develops in stages, as drugs are developed in phases, but in practice, the development may not be linear, stages may sometimes overlap, and not all stages are always required to develop a procedure.

Recently, the Food and Drug Administration launched a consultation on trial design in the localized prostate cancer space, specifically with respect to trial design that might lead to approval of novel therapeutics in localized prostate cancer treatment [31]. The panel included urologists, medical oncologists, radiation oncologists, patient representation, Food and Drug Administration committee members, and industry. The official transcript of the workshop has not been released yet, but during the discussion, it was acknowledged that the assessment of focal therapy in prostate cancer using a given source of energy against standard treatment is more complicated than the assessment of other new technologies in other fields. Indeed, as standard treatments include radical whole-gland treatments, using surgery or radiotherapy, the process of evaluation needs not only take into account the new technology but also the novel approach. Although no definitive conclusion on the ideal design of 1 trial aiming to lead to approval of novel devices was achieved, several issues were raised in terms of specific aspects that should be considered by researchers when structuring these interventional trials.

Nonetheless, we now consider the specific aspects of patient population, intervention, comparator, outcomes, and trial design that are debated in the field.

#### Patient population

According to level 1 evidence arising from the PIVOT trial, patients at low risk do not benefit from active treatment, at least after a median follow-up of 10 years [32]. As a result, many have strongly advised that this population should not be targeted by new technologies. Certainly, although at present most of these men are being treated rather than managed on a program of active surveillance, such an argument may not be a clear justification for focal therapy in this population or for that matter, approving new technologies that use this group within regulatory trials. Equally, it might be questionable to target patients at very high risk, as there is definitive evidence that these patients do benefit from radical treatment and may even require multimodal therapy [32]. Therefore, patients at intermediate and those with low-volume/burden organ-confined high-risk disease seem to be the ideal target population for a novel intervention that attempts to reduce the harms of current treatments but retain the clear benefits that radical treatments confer on prostate cancer mortality [32]. This is a significant shift as focal therapy has been usually considered an alternative to active surveillance, which essentially includes patients at low risk who today are arguably safely managed by active surveillance in the medium and long term [5].

#### Defining the intervention

There is no agreement as to the boundaries and extent of tissue ablation that constitutes focal therapy [33]. Many researchers assert that every approach that is able to spare tissue to whatever extent should be considered a “focal approach” [34]. Others argue that *true* focal therapy is the ablation of an imaging-derived or biopsy-derived target with a certain margin (yet to be defined) and that the indiscriminate ablation of part of the prostate including noncancerous tissue should rather be called “subtotal ablation” [33], [35]. Although this debate remains open and of rather limited interest to most physicians, researchers evaluating a focal approach should clearly apply and report the ablation strategy along with the treatment-specific parameters to make their findings as clear as possible.

#### Comparator

If men at intermediate and high risk represent the target population, the comparator of the index intervention should be the current standard, which are radical treatments, either whole-gland radiation therapy or prostatectomy. Although it is still unclear which of these 2 treatments has the best oncological or functional outcome because no high-quality comparative study has been successfully completed, the toxicity profile is different, with surgery having more genitourinary issues in contrast with radiation therapy having more bowel consequences; however, both show remarkably similar rates of all these toxicities [36], [37]. Furthermore, the time point at which the toxicity after treatment appears is different with surgery and radiation therapy, immediate versus delayed, respectively [36]. Both treatments could be chosen in a very pragmatic manner so that focal therapy is compared with “radical therapy” as a class intervention, but it may be important to choose only one of these interventions to diminish the confounders and to have consistent outcomes across the 2 arms of an RCT, especially if mortality is not chosen as the primary outcome.

#### Outcomes

Cancer-related treatments are usually compared using primary measures based on overall or cancer-specific survival. However, as the natural history of prostate cancer is prolonged, a new trial using this outcome would require a minimum follow-up of approximately 10 to 15 years to show any significant difference between 2 groups assigned to 2 different active treatments and might even need to be powered as an extremely large noninferiority trial [32]. As a surrogate, in traditional whole-gland treatments, PSA kinetics have been used with different thresholds according to each kind of therapy to allow comparisons between different approaches with the same technique, or among different techniques. At present, the use of PSA kinetics in focal therapy is not validated especially as it is difficult to derive a PSA measure that adjusts for ablated cancer/tissue and nonablated cancer/tissue [34]. Therefore, pragmatic outcomes—such as local cancer-control measures and toxicity-related outcomes—seem the only current feasible measures to allow comparison between focal therapy and traditional whole-gland treatments. Local cancer control should be used as a composite outcome, including the need for additional local or systemic treatment and the presence of clinically significant residual disease as determined by histological verification using tests with accurate negative predictive value or by mpMRI if this test would be validated in monitoring after focal therapy. Furthermore, toxicity-related outcomes and quality-of-life measures can allow a standardized and reliable measure of the effect difference of 1 treatment over another. This pragmatic outcome seems extremely important in treatments for prostate cancer given the actual imbalance between harms and benefit. Finally, different approaches and techniques should be challenged by a cost-utility perspective. In fact, as current procedures are increasingly challenged from an economic point of view, in the very near future, the efficacy point of 1 treatment over another is likely to be proportioned with its cost. In the UK, this is required by both trial funders and health care funders.

### Trial design

Various designs can be used to evaluate a new device or intervention.

#### Cohort single-arm studies

High-quality large multicenter single-arm prospective development trials may probably lead to regulatory approval of a new technology, which does not necessarily translate into wider dissemination [38]. Indeed, this kind of trial is recommended by the IDEAL guidelines when researchers are assessing a new technology in stage 2b as it allows testing the effectiveness of a technology in an early phase [27]. Furthermore, this kind of trial has some advantages as compared with comparative trials as it is less demanding in terms of cost, it allows the participation of many centers, and it allows an enhanced recruitment owing to the absence of randomization. Nevertheless, to permit the adoption of a new technology as standard of care, comparative trials might still be necessary following this stage, depending on the degree of patient and physician equipoise remaining.

#### Comparative effectiveness research

To assess the superior effectiveness of a new technology against the standard of care, and therefore change the current practice, the IDEAL guidelines suggest the use of traditional explanatory RCTs, pragmatic RCTs, or alternative designs [27]. The US National Institute of Health definition of comparative effectiveness research is “*comparative effectiveness research is the conduct and synthesis of research comparing the benefits and harms of different interventions and strategies to prevent, diagnose, treat and monitor health conditions in “real world” settings*” [39].

In localized prostate cancer, RCTs have been difficult to recruit to with various trials closing prematurely owing to lack of recruitment arising from lack of physician or patient equipoise, or both [40], [41], [42]. Yet, rather ironically, most physicians and patients would agree that novel interventions in this disease state are very much needed. Therefore, if traditional explanatory RCTs are difficult to carry out, pragmatic designs should be used to provide evidence of effectiveness or ineffectiveness of focal therapy. From a methodological point of view, trials are generally distinguished in explanatory and pragmatic manner. Explanatory trials address the question whether a given procedure can work under ideal conditions in a very selected study population, whereas pragmatic trials answer the question whether a given procedure does actually work in the “real world” using a population with broad entry criteria [43]. In other words, explanatory trials have high internal validity but may have limited external validity, whereas pragmatic trials may incorporate more systematic errors but usually allow wide generalization. As pragmatic trials have easier study design, they are more practicable. For instance, some of the most challenging features to apply in explanatory trials evaluating surgical procedures—such as blinding, strict randomization process, and formal randomization in the same unit—are more flexible in pragmatic trials. Cohort multiple RCTs, cluster RCTs, randomized trials by referral, and randomized consent designs, in which consent is asked after randomization, are all pragmatic trials with specific characteristics that can provide high levels of evidence for the effectiveness of a treatment compared with another [26], [44], [45].

Alternative comparative nonrandomized trials are also feasible [26]. The main advantage of these trials is that they are structured to be feasible in the real world within the actual health care delivery systems. The effectiveness of 1 intervention against the current standard can be assessed by comparing the outcomes of parallel cohorts with nonrandomized allocation [27]. In this setting, the selection bias might be limited by matching patients with similar baseline characteristics across the 2 cohorts. Furthermore, controlled interrupted-time series studies are alternative designs that allow evident comparison of the interventional group with a control group, but do have certain selection biases [27]. Finally, tracker trials in which clinicians allocate patients to different arms based on their own judgment can also allow wide generalizability, but clearly the results may be affected by systematic errors [46]. In conclusion, although these alternative trials are unquestionably more deliverable than the traditional ones, it should be pointed out that they do retain certain selection biases regardless of the methodological adjustment employed. Without randomization, significant selection bias might occur that is difficult to eliminate by matching or statistical adjustment. These include tumor-related parameters and difference in risk of competing mortality in men choosing active surveillance, focal therapy, radical prostatectomy, or external beam radiotherapy. Within the same risk category, men undergoing radiotherapy may have higher risk tumors and higher competing risks than patients undergoing prostatectomy. As a result, large sample sizes and long follow-up periods are often needed to detect the relatively small overall survival differences.

## Conclusions

New technologies might be the answer to counterbalance the shortcomings of the current prostate cancer pathway, from diagnosis to treatment. However, high-quality evidence for the superiority of these new technologies in terms of accuracy, efficacy/effectiveness, and cost-effectiveness is needed to drive the change in the current paradigm, and to allow their dissemination, if truly found to have clinically meaningful positive attributes over the standard pathway. The alternatives to conducting and facilitating high-quality studies are equally grave. The first error is to reject the novel alternatives although they may actually improve the lives of our patients. The second error is to accept the novel alternatives when in fact they are harmful to our patients. High-quality trials that are deliverable in a cost-efficient and timely manner are urgently needed for the benefit of all men who are concerned about having prostate cancer or are currently entangled in making treatment choices between the exhaustive list of therapeutic options available to them.
